# Supernumerary Teeth in the Anterior Maxilla of Non-Syndromic Children and Adolescents: A Retrospective Study Based on Cone-Beam Computed Tomography Scans

**DOI:** 10.3390/pediatric17030052

**Published:** 2025-04-30

**Authors:** Antonis Lykousis, Ioanna Pouliezou, Nikolaos Christoloukas, Aliki Rontogianni, Anastasia Mitsea, Christos Angelopoulos

**Affiliations:** 1 Department of Oral Diagnosis & Radiology, School of Dentistry, National and Kapodistrian University of Athens, 2 Thivon Str., 11527 Athens, Greecenchristoloukas@gmail.com (N.C.);; 2 Medical Research Methodology Unit, School of Medicine, Aristotle University of Thessaloniki, 54124 Thessaloniki, Greece; pouliezou.ioanna@gmail.com; 3 Division of Dental Technology, Department of Biomedical Sciences, University of West Attica, 12243 Athens, Greece; alikironto@yahoo.gr

**Keywords:** supernumerary teeth, CBCT, maxilla, non-syndromic, children

## Abstract

*Background/Objectives:* The aim of this retrospective study was to investigate the supernumerary teeth located in the anterior region of the maxilla of non-syndromic Greek children and adolescents, as well as their possible correlation with demographic characteristics and radiographic findings. *Methods:* The study sample comprised cone-beam computed tomography (CBCT) scans from 224 children and adolescents aged up to 18 years. The following parameters were studied: location of supernumerary teeth in the anterior maxillary area, their morphology, their relationship to adjacent anatomical structures and adjacent teeth, and potential implications. *Results:* Out of the 224 cases 26 (11.6%) presented supernumerary teeth. There was higher prevalence in males than females (61.5% versus 38.5%, respectively). Among the 26 participants diagnosed with supernumerary teeth, one supernumerary tooth was found in 80.8% of children/adolescents, while 19.2% had two supernumerary teeth. The vast majority of supernumerary teeth were impacted (92.3%), and their morphology in 57.7% of cases was conical. A total of 38.5% of cases had normal orientation, 26.9% inverted orientation, 19.2% horizontal orientation, and 15.4% other. The localization was palatal in 84.6%, and the area of localization for 50% of cases was the midline. *Conclusions:* The prevalence of supernumerary teeth in the studied sample of Greek children and adolescents was 11.6% and tended to appear as single, impacted, conical, and with normal orientation. However, these results should be interpreted with caution, due to the limitations in the sampling strategy and the restricted generalizability of this study. The need for further research to enhance broader applicability for different populations is highlighted. These findings are instrumental for a more comprehensive understanding of the prevalence of supernumerary teeth, contributing to more accurate and individualized dental treatment planning in children and adolescents. This will help to avoid future issues in the patient’s dentition.

## 1. Introduction

A supernumerary tooth is defined as any dental or odontogenic structure that exceeds the normal number of 20 deciduous or 32 permanent teeth and can occur in any region of the dental arch [1,2,3]. Supernumerary teeth are regarded as one of the most common developmental anomalies [3] and can be found as an incidental radiographic finding. They may be single or multiple, located in the upper or lower jaw or both, unilaterally or bilaterally. They may be impacted or may have erupted into the oral cavity, in one or both jaws. They usually occur in individuals suffering from a disease or syndrome, while they occur more rarely in individuals with a clear medical history [1,4]. Multiple supernumerary teeth are usually related to conditions or syndromes, such as cleidocranial dysostosis, familial adenomatous polyposis (FAP), Gardner’s syndrome, Rubinstein–Taybi syndrome, Ehlers–Danlos syndrome type III, Nance–Horan syndrome, Ellis–van Creveld syndrome, oculofaciocardiodental (OFCD) syndrome, and cleft lip and palate [3,5,6]. In contrast, individuals without associated diseases or syndromes present a single supernumerary tooth [1,4,6].

The etiology of supernumerary teeth is still ambiguous. Several scientific theories have been proposed to explain the development of supernumerary teeth, such as the dichotomy of the tooth bud, hyperactivity of dental lamina, atavism, and a combination of genetic and environmental factors [7,8,9,10,11]. However, a theory explaining the exact justification and mechanisms of the formation of supernumerary teeth has not been published yet [8,10].

The prevalence of supernumerary teeth is greater in the permanent dentition than in the primary dentition. Previous studies have demonstrated that the prevalence of supernumerary teeth vary from 0.3% to 3.2% for permanent dentition [6,12,13,14] and from 0.07 to 0.6% for primary dentition [15,16], respectively [17,18]. No gender predisposition is observed in the primary dentition. In contrast, in the permanent dentition, the incidence of supernumerary teeth is higher in males than females, presenting a ratio of 1.18:1 to 4.5:1 [5,10,14,19,20].

Supernumerary teeth are often asymptomatic; however, they can lead to various complications. These may comprise impaction, rotation, delayed or ectopic eruption, and displacement of adjacent permanent teeth. Additionally, they can contribute to midline diastema, crowding, root resorption, or disruption of root development in neighboring teeth. In some cases, cyst formation can occur, resulting in secondary alveolar bone resorption [21,22]. Furthermore, supernumerary teeth may cause abnormal root angulation or dilaceration, posing challenges for orthodontic treatment [17]. In rare instances, they can erupt ectopically into the nasal cavity, leading to nasal obstruction or discomfort [17]. They are most commonly located in the anterior region of the upper jaw (pre-maxilla), are conical in shape, and often delay or even hinder the eruption of adjacent teeth [4,5,6]. Therefore, treatment of the supernumerary teeth depends on their type and location, as well as on their immediate potential effect on the permanent teeth. Surgical removal of supernumerary teeth is the treatment of choice in symptomatic, complicated cases and is performed mainly before the start of orthodontic treatment [2,21]. In certain situations, repositioning a supernumerary tooth into the dental arch may be considered an alternative to extraction. This approach depends on factors such as the tooth’s morphology, position, and the overall treatment objectives [17].

The evaluation of the findings resulting from panoramic radiographs has been a valuable tool in routine dental practice. However, with technological development and the introduction of three-dimensional (3D) imaging in the dentomaxillofacial area through cone-beam computed tomography (CBCT), the use of the information provided by two-dimensional (2D) imaging techniques for the detection of supernumerary teeth is considered inaccurate [22]. CBCT is superior to 2D radiographic techniques, as it allows for the detailed imaging of bone and dental structures, as well as their pathology. The information provided on spatial relationships and proximity with neighboring anatomical structures is important for the preoperative evaluation of whether or not to extract supernumerary teeth, optimal treatment planning, and risk assessment of surgery [23]. In cases of surgical extraction of impacted teeth, it has been reported that the construction of a surgical splint using dental CBCT imaging data contributes to the precise localization and more atraumatic surgical removal of supernumerary teeth [24].

The existing body of evidence has demonstrated the diverse prevalence of supernumerary teeth in certain ethnic or age groups and some ethnic- and age-specific characteristics of supernumerary teeth among diverse populations [15,25]. The necessity of conducting research for population-specific reports on the prevalence and characteristics of supernumerary teeth has recently gained awareness. As a result, there is a growing number of relevant published investigations for various populations. However, the prevalence, localization and possible clinical implications of supernumerary teeth in the Greek population of children and adolescents based on the analysis of CBCT scans has not been established. To our knowledge, this study is the first investigation of supernumerary teeth using CBCT scans of a Greek sample aged between 8–18 years old. The results of the current study may provide further insight to the clinicians who treat non-syndromic children and adolescents with supernumerary teeth in establishing refined comprehensive treatment protocols.

The primary aim of this study is to evaluate the supernumerary teeth located in the anterior region of the maxilla of Greek children and adolescents (non-syndromic) and their differences in demographic features (gender and age). A secondary objective is to investigate the correlation of supernumerary teeth with both demographic features and CBCT findings.

## 2. Materials and Methods

### 2.1. Study Design, Protocol, and Ethics

We conducted a cross-sectional study using a retrospective approach. The study was performed according to the ethical standards of the Declaration of Helsinki and the guidelines of the Strengthening the Reporting of Observational Studies in Epidemiology (STROBE) statement [26]. The protocol of this study was approved by the Research and Ethics Committee of the School of Dentistry of the National and Kapodistrian University of Athens (no. 590/23.05.2023). The approved protocol was followed without deviation from its original design. Informed consents were not sought from the participants of the study, since it was based on a retrospective design, and they had already consented to research participation prior to their radiographic examination. Thus, we used records already obtained from the clinic of the Department of Oral Diagnosis and Radiology, School of Dentistry, National and Kapodistrian University of Athens, Greece. All CBCT scans were conducted according to the ALARA (as low as reasonably achievable) principle, with an indication that justified exposure to radiation. Prior to any analysis, the CBCT images were anonymized so that participants’ identities could not be specified.

### 2.2. Study Setting

The present study retrospectively analyzed 224 CBCT scans carried out between 1 January 2015 and 30 November 2022. All of the scans were conducted at the Department of Oral Diagnosis and Radiology of the School of Dentistry, National and Kapodistrian University of Athens.

### 2.3. Study Population

The study sample comprised available CBCT scans of all young patients who sought dental care at the university’s clinic until November 2022. The reasons that justified patients’ radiographic examinations were the following: orthodontic assessment and planning, pre-surgical orthognathic planning, evaluation of facial trauma or fractures, assessment of temporomandibular joint disorders, impacted third molars, unerupted permanent teeth, evaluation of airway and sinus-related conditions, assessment of sleep apnea or breathing issues, and detection and monitoring of any cysts, tumors, or other abnormal growths in the jaw or facial bones. Initially, 3.914 patients’ records were screened based on the eligibility criteria for their inclusion in the study.

#### Eligibility Criteria

The inclusion criteria were: (1) patients under 18 years of age, (2) without any systemic diseases, (3) no tooth extractions, and (4) CBCT imaging that captured the maxillary bone (hard tissue). Exclusion criteria included: (1) patients aged 18 or older, (2) manifestation of any systemic disease, (3) tooth extractions performed, and (4) CBCT scans that did not adequately visualize the maxillary anterior region. After applying these criteria, a final sample of 224 CBCT scans was selected for the study.

### 2.4. Study Outcomes

The primary outcome was to assess the differences in demographic features (gender and age) between non-syndromic children/adolescents with supernumerary teeth in a Greek population. Secondary outcomes included examination of the correlation of supernumerary teeth with both demographic features and CBCT findings. In particular, the following parameters were studied: a. location of supernumerary teeth in the anterior maxillary area, b. their morphology, c. their relationship to the adjacent anatomical structures and adjacent teeth, and d. potential implications.

### 2.5. CBCT Scans’ Evaluation, Data Collection, and Extraction

The scans were ordered for therapeutic reasons of the patients and not for the objectives of this study. All CBCT images were performed according to the ALARA principle, with an indication justifying radiation exposure. The same equipment (CBCT scanner New Tom 5G, Verona, Italy) was employed during the radiographic examinations of all study participants. DICOM files with all related CBCT information for every individual patient were collected and imported into the NNT software program (version 4.6, New Tom, Verona, Italy) for further assessment.

All CBCT images were screened independently by two observers (A.L. and N.C.) in the same examination setting under standardized conditions. In the event of disagreement between the observers, consensus was reached by discussion. Ambiguous findings and obvious inter-observer disagreements were thoroughly discussed with a third observer, an experienced oral and maxillofacial radiologist (A.M.), before the final decision.

Data from 224 children and adolescents with maxillary CBCT scans were collected after thorough examination and extracted in Microsoft© Excel© 365 software (version 16.80; Microsoft Corp., Redmond, WA, USA) form designed for this study. The following details were systematically recorded for each of the 224 CBCT scans of the study sample:

a. Sex and age (in years) of each subject.
b. Imaging technique (modality) used to identify the supernumerary teeth.
c. Number, size, shape, and location of the supernumerary teeth detected in the maxillary anterior area.
d. Level of impaction of each supernumerary tooth (impacted, semi-impacted, fully erupted).
e. Spatial orientation of supernumerary teeth in the sagittal plane.
f. Relationship and proximity of supernumerary teeth to adjacent anatomical structures and adjacent teeth.
g. Implications of supernumerary teeth on the dental arch.

### 2.6. Statistical Analysis

Two observers (A.L. and N.C.) systematically reviewed and analyzed all descriptors of demographic and clinical characteristics. Central tendency and dispersion measures were calculated, in addition to frequency and relative frequency distributions. The research questions in this study were mainly:

a. To inquire about the differences in demographic features (gender and age) between children/adolescents of supernumerary teeth;
b. To investigate the correlation of supernumerary teeth with both demographic features and CBCT findings.

The chi-square (*χ*^2^) test was used to test research hypotheses regarding the relationship between two qualitative variables. In case the conditions of the chi-square test were not satisfied, a Fisher’s exact test was employed instead. Continuous variable age was investigated for normality using the Shapiro–Wilk test. The variable was normally distributed in both numbers of the supernumerary teeth groups, one and two (*p*-value = 0.120 and *p*-value = 0.777 > 0.05, respectively). To compare the mean values, a two-tailed *t*-test was performed for independent samples. The *z*-test was used to compare proportions.

The reported *p*-values were based on two-tailed tests. Particularly, *p*-values less than 0.05 were considered statistically significant results. The statistical software program SPSS v.26 (IBM Corporation, 2019 Armonk, New York, NY, USA) was used to conduct the statistical analysis.

## 3. Results

The demographic features’ distribution of children and adolescents with or without supernumerary teeth are demonstrated in Table 1. Frequencies and respective percentages were provided for categorical (qualitative) variables. Continuous variables were described by mean value and standard deviation value (SD). On the basis of statistical analysis, it was reported that there were no statistically significant differences in sex or age variables among the individuals (*p* > 0.05). A total of 26 individuals (11.6%) were diagnosed with supernumerary teeth in the examined sample.

Descriptive analysis of the sample’s demographic data reporting supernumerary teeth (n = 26) and their radiological findings are presented in Table 2. In the sample, 61.5% of the sample were males, while 38.5% were females. The average age of the study sample was 14.6 years (SD = 2.5 years) and ranged from 8 to 18 years old.

**Figure 1:**
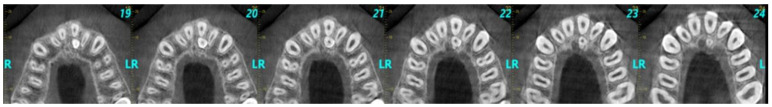
Axial images of the CBCT scanning (slice thickness 1 mm). The supernumerary tooth is located palatally along the midline and the left central incisor (R: right, L: left).

**Figure 2:**
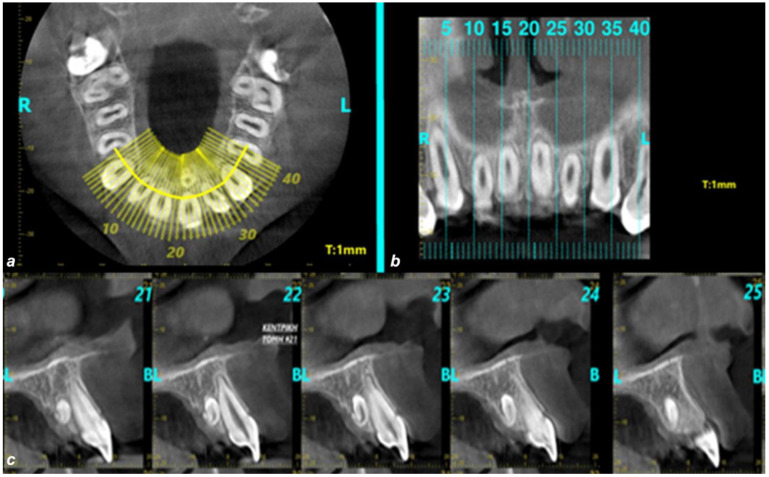
Sagittal images of the CBCT scanning (slice thickness 1 mm). The impacted supernumerary tooth (**a**) presents an inverted orientation (R: right, L: left.), (**b**) its root apex exhibits a pronounced palatal curvature due to limited space (R: right, L: left.), and (**c**) it is in contact with the palatal surface of the left central incisor without causing its resorption (B: buccal, L: lingual). The radiographic findings are indicative of an atypical supernumerary incisor.

**Figure 3:**
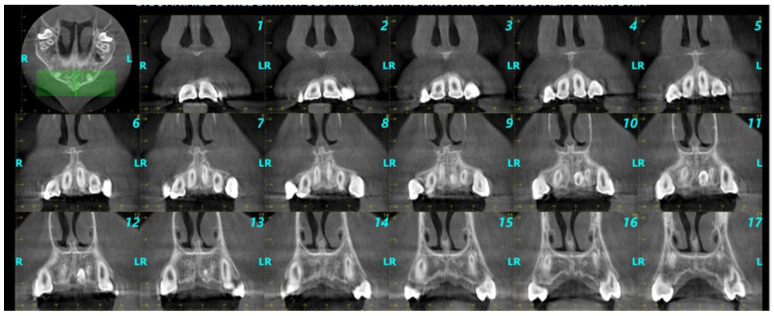
Coronal images in the anterior region of the maxilla (slice interval 1 mm, R: right, L: left, each green line refers to a coronal slice).

Radiological findings are depicted in Figure 1, Figure 2 and Figure 3. Among the participants who were diagnosed with supernumerary teeth (n = 26) in our sample (non-syndromic children/adolescents), it was observed that 80.8% presented with a single supernumerary tooth and 19.2% with two supernumerary teeth. The majority of supernumerary teeth were impacted (92.3%), with conical morphology being the most prevalent (57.7%). In terms of orientation, 38.5% were normally positioned, 26.9% were inverted, 19.2% were horizontally oriented, and 15.4% fell into other categories. The most common location was the palate (84.6%), particularly in the midline region (50%) (Figure 1, Figure 2 and Figure 3).

Based on sagittal classification, 40% were situated near the cervical region of adjacent teeth, with implications for neighboring teeth observed in 26.9% of cases.

**Figure 4:**
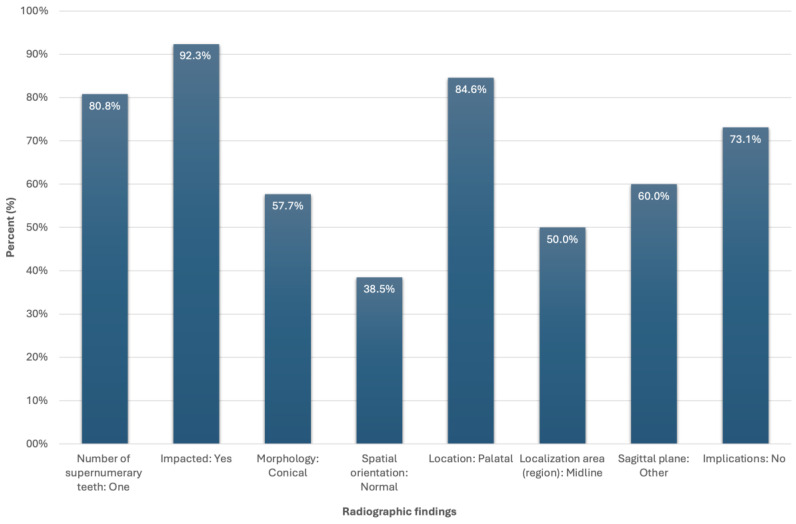
Distribution of the sample’s radiographic findings.

Therefore, it emerged that a 14.6-year-old adolescent male in whom a single conical-shaped supernumerary tooth (normal form) was located palatally and specifically in the midline was the typical profile of our sample’s participant. These characteristic radiographic findings are also depicted graphically in Figure 4.

**Figure 5:**
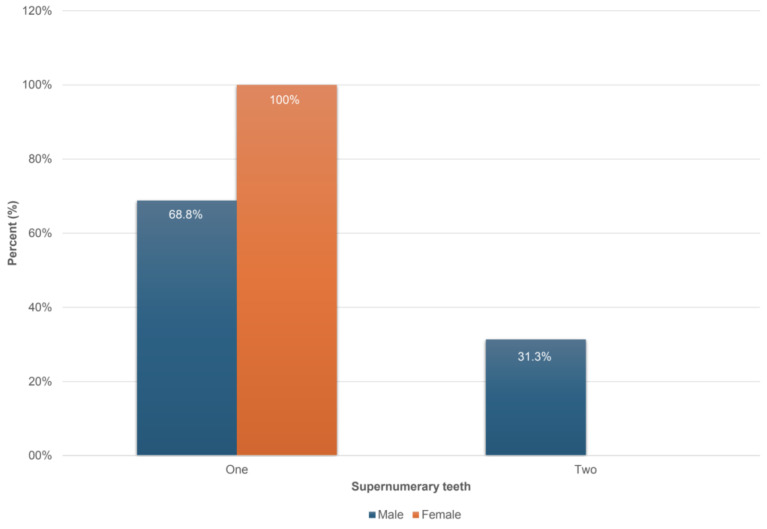
Bar graph of the supernumerary teeth distribution by sex.

Data depicted in Table 3 revealed a significant but weak correlation among the supernumerary teeth number and the demographic features data combined with the CBCT radiographic findings in the studied sample. The quantitative variable of supernumerary teeth was correlated statistically significantly (*p* = 0.049 < 0.05) with the sex variable. Additionally, two supernumerary teeth presented in a greater percentage (31.3%) in males compared to females. The difference in proportions was also statistically significant (*z*-test, *p*-value = 0.049 < 0.05). The results are also graphically demonstrated in Figure 5. No significant differences in the age variable were detected based on the presence or number of supernumerary teeth.

Regarding radiographic findings, the two supernumerary teeth were more likely to be impacted, conical in shape, located in the maxillary palate along the midline, and near the cervical region when compared to a single supernumerary tooth, but these differences were not statistically significant (*p* > 0.05).

## 4. Discussion

### 4.1. Summary of Evidence

Supernumerary teeth are defined as odontogenic structures or well-formed teeth, the number of which is greater than normal in any area of the dental arch. They are considered to be developmental anomalies and are an incidental radiographic finding in the everyday clinical practice of pediatrics, general dentistry, and oral–maxillofacial surgery [3,27]. The prevalence of supernumerary teeth varies from 0.3% to 0.8% in the primary dentition and from 1.5% to 3.5% in the permanent dentition [17,18,28,29]. They appear both as single/multiple and unilateral and/or bilateral of the midline. Detection of multiple supernumeraries is relatively rare and is mainly associated with the manifestation of pathological syndromes, such as Gardner syndrome and cleidocranial dysostosis [30]. The very limited number of publications in the Greek and international bibliography has demonstrated that the location of supernumerary teeth in patients with no medical history is a rare entity [5,31]. Although they are found everywhere in the oral cavity, they show an increased tendency to appear in the anterior region of the maxilla of the primary and permanent dentition [32,33]. In general, dental anomalies are more frequent in the maxilla and mainly involve the anterior teeth, which is probably due to the evolutionary history of the jaws and ontogeny. Moreover, this can be attributed to the more complex development of maxilla and its tight interaction with other cranial structures. Specifically, supernumerary teeth were found, based on a recent study, predominantly in the maxilla rather than the mandible (80.8% vs. 19.2%), while all supernumerary teeth appeared in the maxillary anterior region (100%) [34]. Appearance of the supernumerary teeth in the anterior maxilla has been explained as resulting from atavism (phylogenetic reversion), which is the reappearance of an ancestral condition or type. This hypothesis proposes a reversion to an ancestral human dentition that contained a larger number of teeth [34,35].

The aim of the study was to record and study the supernumerary teeth found in the anterior region of the maxilla in non-syndromic children and adolescents, as well as their possible correlation with demographic characteristics and radiographic findings.

In our study, it was observed that 80.8% of children/adolescents have a supernumerary tooth, and the vast majority of them are impacted (92.3%), a finding that is in agreement with the study by He et al. [36]. In their study [36], the potential relationship between the existence of supernumerary teeth and subsequent impaction of the anterior regions of the maxilla in mixed dentition was investigated in a sample of 294 patients (220 males-74 females, age range 6–12 years). The parameters that were examined and ultimately emerged as statistically significant for the sample were the following: molariform and odontoma-like supernumerary teeth, coronal supernumerary teeth, normal, and no orientation. The authors argued that an increase in age by one year is associated with a reduced risk of the simultaneous detection of supernumerary and impacted incisors, while an increase in the number of supernumerary teeth by one almost doubles the probability of coexistence with an impacted incisor [36]. It is noteworthy that the prevalence of supernumerary teeth in the mixed dentition of the sample was three times higher in males than in females. Also, the reported incidence of supernumeraries in the retrospective study was excessively high (23.1%) and deviated significantly from the average found in the international literature (0.3% to 0.8% in primary dentition) [19,20].

The systematic evaluation of computed tomography scans and statistical analysis of the data in our study revealed that the morphology of supernumerary teeth in 57.7% of cases was conical, while 38.5% of cases had a normal orientation in space, 26.9% inverted, 19.2% horizontal, and 15.4% other. Similar rates were also published in the study by Shekhar [37] and Kim et al. [38].

In the retrospective study by Shekhar [37], a total of 11,200 children, aged 3–12 years, who sought dental treatment at the Pediatric Clinic of Jazan University during the period 2007–2010 were examined. Finally, 212 individuals were selected, according to the inclusion and exclusion criteria. The aim of their investigation was to capture the characteristics and distribution of the supernumerary teeth in the anterior region of the maxilla, both in primary and mixed dentition, in a young population originating from India. The participants were divided into two groups based on the type of dentition. The final diagnosis and classification of supernumerary teeth was performed by taking panoramic and bitewing radiographs. Although single supernumerary teeth were identified in the majority of cases (84.9%), the percentage of >2 supernumeraries was only 14.6%, significantly lower compared to similar studies [39,40]. Out of a total of 246 supernumerary teeth, 68.7% were conical in shape, while 20.7% were cylindrical in shape, and the remaining 10.6% were various variations thereof. This finding is consistent with results from similar studies [7,41,42]. Shekhar [37] reported an overall prevalence of 1.9% in his study, which is marginally higher than a previous study in a similar population [43], and a relatively high prevalence in males than in females (1.7:1), a result that is not consistent with the majority of published articles in international literature, which support at least a double ratio of males to females [12,14,44].

Kim et al. [38] determined the position of the incisors in a three-dimensional plane (frontal–sagittal–transverse) using CBCT and panoramic radiographs as a complementary mean. The application of the inclusion–exclusion criteria limited the initial sample of 444 individuals to 293 participants (age range 4–10 years, with no history of orthodontic treatment), with the total number of incisors reaching 383. The CBCTs were evaluated using the appropriate software (Ez3D plus software, version 1.2.6.23, Vatech, Hwasung, Republic of Korea). Statistical analysis did not reveal a statistically significant correlation between the morphology of the mesiodens and their complications regarding the resorption of adjacent teeth, although these were observed in 33.7% of cases. The most frequent location of the interdental teeth was palatal and close to the tooth cervix (61.9%), with conical being by far the most prevalent type of morphology (86.4%) [38].

Anegundi et al. [45] reported a similar prevalence rate of conical supernumerary teeth in a pediatric population of Indian origin (82.28%), a finding that is consistent with several other studies [46,47]. In their retrospective study [45], 63,569 patients, up to 14 years of age, were initially examined, and their final sample consisted of 790 individuals after applying the eligibility criteria. In 92.53%, the supernumeraries were located in the anterior region of the maxilla, a finding that is consistent with similar population studies [19,48]. Their study demonstrated an increased ratio of males to females (1.5:1), which is consistent with previous studies performed by Gábris et al. [48] (1.4:1), Brook [49] (1.4:1), and Fernández-Montenegro et al. [20] (1.4:1). In contrast, the studies of Giancotti et al. [50] and Berrocal et al. [13] reported no significant difference between sexes. The present investigation also reported a higher prevalence in males than females, 61.5% versus 38.5%, respectively, indicating a male to female ratio of 1.59:1. The studies by Ata-Ali et al. [17] and Brook et al. [18] reported that the incidence is greater in males than in females, with a sex ratio ranging between 1.18:1 and 4.5:1, respectively.

Supernumerary teeth can appear as single or multiple entities in any area of the dental arches [19,51]. Previous studies have reported that among individuals with supernumerary teeth, 76–86% have a single supernumerary tooth, while 14–24% have more than one [19,52]. Our study supported these findings, as 80.7% of the affected individuals (n = 26) had a single supernumerary tooth, while 19.2% exhibited two.

In our study, the location of supernumeraries was palatal in 84.6%, and the area of their occurrence was the midline in 50% of cases. Ma et al. [53] indicated higher rates of supernumerary teeth in the maxilla (94.86%), and the most common location was between the central incisors (mesiodens) (2312 cases and a rate of 88.92%). In their prospective study, 2768 CBCT images of 1984 individuals aged 6–17 years who had been examined at the Affiliated Stomatological Hospital, Nanjing Medical University from June 2012 to December 2018 were evaluated. Patients with a previous history of orofacial developmental abnormalities, surgical extractions, or syndromes related to supernumerary teeth were excluded from the study [53]. Conical supernumerary teeth had the highest frequency (2194 teeth out of a total of 2768) distributed in the incisor region (2021 among the central incisors and 112 in the lateral incisor region), with 35 in the canine region.

Regarding the dental implications, we reported an overall moderate rate of 26.9% in the total sample and a higher prevalence of implications (40%) in cases of two supernumerary teeth rather than one supernumerary tooth (23.8%). In the cohort study by Ma et al. [53], the most frequent complications in adjacent permanent teeth (associated with a supernumerary tooth) were their ectopic position (336 teeth), their rotation and change in orientation (151 cases), and their impaction (150 teeth), with a clear predisposition to occur in the anterior region. In addition, 15 supernumerary teeth were associated with cystic lesions in the anterior region of the maxilla (9 in the central incisor region, 4 in the lateral incisor region, and 2 in the canine). It is worth mentioning that root resorption of adjacent teeth was observed in only five cases and especially in the posterior region [53].

The presence of mesiodens is a common and incidental radiographic finding in childhood; its prevalence varies among ethnic groups and is observed to be higher in Asian populations (on the order of 3% or higher) compared to Caucasians [25,51]. In addition, there is a clear male to female predominance, with a ratio of 5.5:1 in the Japanese population and 3.1:1 to 6.5:1 in a child population of Hong Kong origin [5,54]. Interdental spaces develop both unilaterally and bilaterally in the anterior region of the maxilla [55,56] and can result in potential complications, such as delayed growth or delayed eruption of the adjacent tooth, crowding, impaction and resorption, ectopic eruption, wide interdental space, and cystic lesion [40].

The appropriate time for mesiodens extraction is a field of controversy in the dental scientific community. Some studies support that any intervention in the anatomical area should follow the completion of the formation of the roots of the adjacent teeth; essentially, the individual must have completed the 10th year of their age (8–10 years) [57]. In contrast, other studies suggest removing mesiodens as soon as possible, immediately after their diagnosis, in order to avoid possible complications, such as extensive alveolar bone removal in future extraction of the impacted area [7]. Although all theoretical approaches are distinguished by undeniable advantages and disadvantages, most published relevant studies are case-series presentations, and only one is a prospective study (cohort study) [2].

In agreement with other studies, supernumerary teeth show an increased predisposition in males, compared to females, and especially in Asian populations, compared to Caucasians [5,51]. Regarding morphology, conical-type supernumeraries presented the highest frequency in our study, a result that agrees with previous studies [5,14,58,59]. The incidence of a single supernumerary tooth is higher than their multiple detections in the jaws [14,60,61]. These findings could be attributed to evolutionary changes in the human masticatory system, leading to its rapid and considerable adaptation to changing demands.

### 4.2. Limitations

Limitations of the present study include the relatively small sample size compared to previous reports. Nonetheless, due to the rarity of supernumerary teeth, collecting a large sample can be challenging. In addition, the higher prevalence of supernumerary teeth in our study (11.6%), compared to previous reports (0.3–3.2%), could be attributed to the sampling strategy. We enrolled children and adolescent patients who sought dental or maxillofacial treatment, while in other studies, mainly healthy children and adolescents were involved. Thus, further investigation of the general population is needed. Another consideration for this study is the potential bias attributed to the study sample’s lack of ethnic diversity, as all subjects were Caucasian. Furthermore, the radiographic assessments in this investigation were based on a retrospective approach, which presents another limitation. However, ethical concerns about the radiation exposure of the imaging techniques rendered a prospective study impossible. Therefore, radiographs obtained solely for therapeutic purposes are the only ones allowed, and researchers are not permitted to expose any subjects to radiation for research.

### 4.3. Generalizability (External Validity)

The generalizability of the present study is restricted to the tested sample with regard to the ethnic group. Our database comprised patients from the Greek population examined in a single setting. The range of patients was sufficient: females and males, from young children and adolescents aged up to 18 years old, were included.

### 4.4. Future Research

Further research regarding supernumerary teeth between children/adolescents is still required on an international scale to establish the most effective and population-specific approaches to minimize the effect of this condition on dental health and occlusion, as well as to optimize the quality of care delivered to affected individuals. Therefore, subsequent investigations should examine diverse ethnic groups and include greater sample sizes in order to fully understand the potential correlation of supernumerary teeth with both demographic features and CBCT findings.

## 5. Conclusions

The conclusions derived from this study regarding the supernumerary teeth in the anterior maxillary region of the examined population are the following:

- There is higher prevalence in males.
- Supernumerary teeth appear more frequently as single and impacted, with a conical shape and normal spatial orientation, while their most frequent location is the middle line of the palate.

However, these findings should be interpreted with caution, due to the limitations in the sampling strategy and the restricted generalizability of this study. Hence, the need for further research to enhance broader applicability for different populations is highlighted.

Clinicians should carefully consider the prevalence and typical characteristics of supernumerary teeth when developing effective interceptive treatment plans for potentially severe oral rehabilitation issues. A meticulous evaluation of all diagnostic images and a detailed examination of each anatomical region during the diagnostic assessment are essential for facilitating effective interdisciplinary treatment and for highlighting the potential necessity for future dental care in individuals with supernumerary teeth associated with complications. In addition, early diagnosis and management of such cases of supernumerary teeth will contribute to more accurate and individualized dental treatment planning. This will help to prevent future adverse effects on the patient’s dentition.

## Figures and Tables

**Table 1:** Demographic features according to the presence/absence of supernumerary teeth in the sample’s participants.

Demographic Features	Supernumerary Teeth		*p*-Value
	Yes (*n* = 26)	No (*n* = 198)	
**Sex**	**n (%)**	**n (%)**	
Males	16 (61.5)	99 (50.0)	0.268 ^1^
Females	10 (38.5)	99 (50.0)	
**Age (years: mean value, SD)**	14.6 (2.5)	14.8 (2.6)	0.712 ^2^

SD: Standard deviation. ^1^ *χ*^2^ test. ^2^ *t*-test for independent samples, statistically significant results at *p* < 0.05.

**Table 2:** Distribution of sample’s demographic data.

Demographic Features	Descriptive Statistical Measures
**Sex**	**n (%)**
Males	16 (61.5)
Females	10 (38.5)
**Age** (years: mean value, SD)	14.6 (2.5)
**Radiographic findings**	
**Number of supernumerary teeth**	**n (%)**
1	21 (80.8)
2	5 (19.2)
**Impacted**	**n (%)**
No	2 (7.7)
Yes	24 (92.3)
**Morphology**	**n (%)**
Conical	15 (57.7)
Other	11 (42.3)
**Spatial orientation**	**n (%)**
Normal	10 (38.5)
Inverted	7 (26.9)
Horizontal	5 (19.2)
Other	4 (15.4)
**Location**	**n (%)**
Palatal	22 (84.6)
No	4 (15.4)
**Localization area (region)**	**n (%)**
Middle line	13 (50.0)
No	13 (50.0)
**Sagittal plane**	**n (%)**
Adjacent to tooth neck (cervix)	10 (40.0)
Other	15 (60.0)
**Implications**	**n (%)**
No	19 (73.1)
Yes	7 (26.9)

**Table 3:** Correlation among the supernumerary teeth number and demographic data combined with CBCT radiological findings.

Demographic Features	Number of Supernumerary Teeth		*p*-Value
	One (*n* = 21)	Two (*n* = 5)	
**Sex**	**n (%)**	**n (%)**	
Males	11 (68.8)	5 (31.3)	0.049 ^1,^*
Females	10 (100.0)	0 (0.0)	
**Age** (years: mean value, SD)	14.4 (2.6)	15.2 (1.5)	0.538 ^2^
**Radiographic findings**			
**Impacted**	**n (%)**	**n (%)**	0.646 ^3^
No	2 (9.5)	0 (0.0)	
Yes	19 (90.5)	5 (100.0)	
**Morphology**	**n (%)**	**n (%)**	
Conical	12 (57.1)	3 (60.0)	0.907 ^1^
Other	9 (42.9)	2 (40.0)	
**Spatial orientation**	**n (%)**	**n (%)**	
Normal	7 (33.3)	3 (60.0)	0.506 ^3^
Inverted	5 (23.8)	2 (40.0)	
Horizontal	5 (23.8)	0 (0.0)	
Other	4 (19.0)	0 (0.0)	
**Location**	**n (%)**	**n (%)**	
Palatal	17 (81.0)	5 (100.0)	0.289 ^1^
No	4 (19.0)	0 (0.0)	
**Localization area (region)**	**n (%)**	**n (%)**	
Middle line	9 (42.9)	4 (80.0)	0.1351 ^1^
No	12 (57.1)	1 (20.0)	
**Sagittal plane**	**n (%)**	**n (%)**	
Adjacent to tooth neck (cervix)	7 (33.3)	3 (75.0)	0.119 ^1^
Other	14 (66.7)	1 (25.0)	
**Implications**	**n (%)**	**n (%)**	
No	16 (76.2)	3 (60.0)	0.463 ^1^
Yes	5 (23.8)	2 (40.0)	

SD: Standard deviation. ^1^ *χ*^2^ test. ^2^ *t*-test for independent samples. ^3^ Fisher’s exact test, * statistically significant result at *p* < 0.05.

## Data Availability

The data presented in this study are available upon request from the corresponding author due to the EU General Data Protection Regulation.

## Abbreviations

The following abbreviations are used in this manuscript:

CBCT	Cone-beam computed tomography
FAP	Familial adenomatous polyposis
OFCD	Oculofaciocardiodental syndrome
ALARA	As low as reasonably achievable
